# Formulation and Evaluation of Guggul Lipid Nanovesicles for Transdermal Delivery of Aceclofenac

**DOI:** 10.1155/2014/534210

**Published:** 2014-02-06

**Authors:** Praveen Kumar Gaur, Shikha Mishra, Vidhu Aeri

**Affiliations:** ^1^Department of Pharmaceutics, I.T.S. Paramedical (Pharmacy) College, Muradnagar, Ghaziabad 201206, India; ^2^Department of Pharmacognosy & Phytochemistry, Jamia Hamdard, New Delhi 110062, India

## Abstract

*Context*. Most new drugs have low water solubility and liposome is an important formulation to administer such drugs; however, it is quite unstable and has negligible systemic absorption. *Objective*. Aceclofenac nanovesicles were made using guggul lipid for formulating stable transdermal formulation. *Materials and Methods*. Guggul lipid was formulated into vesicles along with cholesterol and dicetyl phosphate using film hydration method. The formulations were analyzed for physicochemical properties and stability. Then its skin permeation and anti-inflammatory activity were determined. *Results*. Both categories of vesicles (PC and GL) showed optimum physicochemical properties; however, accelerated stability study showed considerable differences. GL-1 was appreciably stable for over 6 months at 4°C. Corresponding gels (PCG-1 and GLG-1) showed *C*_max_ values at 4.98 and 7.32 **μ**g/mL along with the *T*_max_ values at 4 and 8 hours, respectively. GLG-1 inhibited edema production by 90.81% in 6 hours. *Discussion*. PC liposomes are unstable at higher temperature and upon longer storage. The formulation with higher lipid content (GL-1) showed good drug retention after 24 hours and appreciable stability both at higher temperature and for longer duration. Guggul lipid being a planar molecule might be stacked in vesicle wall with cholesterol. *Conclusion*. The composition of the nanovesicle played an important role in stability and drug permeation. Guggul lipid is suitable for producing stable vesicles.

## 1. Introduction

In the present scenario of fast pace drug discovery system, various drug delivery systems are being developed to further enhance the therapeutic efficacy of new drugs as well as to improve upon the side effects of the active ingredient by means of formulation development. Further, it has become important for the drug delivery system to afford controlled drug delivery at the specific site [1]. Generally out of 10 new drugs reported 8 drugs possess very low water solubility thus making it worthwhile to formulate them in lipid based formulations.

Liposome has been an important lipid based formulation indicated for topical and transdermal applications. Structurally, liposome contains an outer mono- or bilayer of molecules surrounding hollow core which serves as storage for the therapeutic agent. Liposomes can accommodate physicochemically different drugs in liposome membrane (hydrophobic) and internal core (hydrophilic) [2–4]. Liposome can enhance the bioavailability and improve elimination of rapidly metabolized drugs, so it has been successfully used as vehicles for controlled drug delivery [5]. Since last three decades liposomes have been studied for various applications; among them transdermal applications are mostly reported [6].

In the literature, the term liposome refers to vesicles containing phospholipid as main lipid component, in particular, phosphatidylcholine; however, the stability and permeation profile for liposome are two important areas needing consideration [6, 7]. Phospholipids are detrimentally affected by hydrolysis and oxidation. Hydrolysis of phospholipids yields lysolecithin which enhances the permeability of liposomes thereby causing leakage of entrapped drug. Physically also, liposomes are affected by lowering pH [8]. Permeation studies *in vitro* have revealed that PC liposome may produce several-fold higher drug concentrations in the epidermis and dermis and lower systemic concentrations when compared to conventional dosage forms [9].

Owing to the above-mentioned drawbacks, there is a need to explore new lipid molecules for formulating into liposome with improved stability and permeation profile. In the present study, we have developed a nanovesicular formulation containing guggul lipid as main lipid component to improve the stability profile of the formulation. Further, the developed formulation was evaluated for its transdermal application since drug administration through skin offers advantages of avoidance of GIT and first pass metabolism and also can improve upon the drugs' side effect, mainly gastrointestinal irritation [10].

Guggul lipid is guggulsterone (4,17(20)-pregnadiene-3,16-dione), which is present in a concentration of 4.0–6.0% in ethyl acetate extract obtained from *Commiphora wightii *(Family: Burseraceae). The guggulsterone is a mixture of E and Z stereoisomers in which Z-isomer is potent antilipidemic. Chemically, guggul lipid is steroid similar to cholesterol; however, it lacks the side chain. Cholesterol is a major component of various lipid based drug delivery systems and it is reported to improve the stability of the liposome [11–14].

Aceclofenac is an analgesic, antipyretic, and anti-inflammatory drug and is indicated in rheumatoid arthritis, osteoarthritis, and ankylosing spondylitis. It acts on COX-2 isozyme to reduce the production of inflammation mediators [15–17]. Aceclofenac has also showed glycosaminoglycan synthesis stimulation in human osteoarthritic cartilage. It shows side effects relating to GIT, liver, and kidney function as well as disturbance of platelets function [16, 18].

It is practically water insoluble, having a molecular weight of 354.19, pKa value 4.7, and log *P* value 1.23 [19]. It is BCS- class II compound for whom oral bioavailability is decided by dissolution rate in GIT. These factors make it appropriate to formulate it in transdermal formulation [16].

In the present study, we have developed a nanovesicle formulation using guggul lipid as main lipid component for Aceclofenac. The formulations were evaluated for physicochemical parameters, transdermal permeation, stability, and anti-inflammatory activity. The selected formulation was compared with an established transdermal aceclofenac formulation (ACE-PROXYVON GEL, aceclofenac 1.5% w/w) in permeation studies. A gel formulation containing plain aceclofenac was also prepared and compared with designed formulation. This study will be useful in devising improved formulations for transdermal application.

## 2. Materials and Methods

### 2.1. Materials

Aceclofenac was the gift sample from Akums Drugs & Pharmaceuticals Ltd., Haridwar, India. Guggul lipid was purchased from Sami Labs Limited, Bangalore, Karnataka, India. Cholesterol (Chol) was purchased from Himedia, Mumbai, India. Phosphatidylcholine (PC) and dicetyl phosphate (DCP) were purchased from Sigma-Aldrich (New Delhi, India). All other materials were of analytical grade. Commercial formulation was ACE-PROXYVON GEL (aceclofenac 1.5% w/w) manufactured by Wockhardt Merind Limited (Wockhardt. Ltd. Enterprise), Mumbai, India.

### 2.2. Methods

#### 2.2.1. Formulation

Lipid film hydration method was used to formulate the nanovesicles as per Table 1. The drug and lipids were dissolved in chloroform. The solvent was evaporated from drug-lipid solution by means of vacuum evaporation (Rotavapor R II, BUCHI India Private Ltd.) to form a thin film on the wall of the flask. Then acetate buffer solution (pH 5.5) was added to the flask to hydrate the film. The setup was stirred at 200 rpm for 1 hour at 25°C and then sonicated using a probe sonicator for 15 minutes at 100 W amplitude obtaining a homogeneous dispersion. The formulation was extruded through a membrane (Immobilon-P Membrane, 0.45 *μ*m pore size, Millipore Pvt. Ltd., New Delhi, India).

This dispersion was passed through sephadex G-20 minicolumn to remove unentrapped drug [6, 20, 21].

#### 2.2.2. Size Determination

Negative staining followed by TEM was employed to estimate the shape and size of formulation. An aliquot of test sample was located over the copper grid followed by phosphotungstic acid (1%). Then test sample was dried at room temperature and analyzed by using TEM (Philips CM-10, acceleration voltage: 100 kV; magnification: up to 450,000x; Cryoattachment).

#### 2.2.3. PDI and Zeta Potential

Zetasizer (Nano-ZS, Malvern Instruments, UK) fitted with a 4 mW He-Ne laser was used to analyze polydispersity indices and zeta potential. The test sample was lyophilized and then suspended in phosphate buffer (5.5). It was then placed into microcentrifuge tube to determine the number of photons (kilo count per second) for analyzing the result.

#### 2.2.4. Entrapment Efficiency

Ultracentrifugation method was employed for determining the entrapment efficiency. An aliquot of formulation was centrifuged at 12000 rpm using ultracentrifuge (Remi C-24 BL with angular R-241 rotor, Remi House, Mumbai, India) and content of drug was estimated separately in the sediment and the supernatant [22]. The entrapment efficiency was estimated as follows:

(1) $$\begin{array}{c} \left[\frac{\left(T-C\right)}{T}\right]\times 100, \end{array}$$

where *T*  is total drug content and *C* is drug content in supernatant [23].

#### 2.2.5. Assay

HPLC method using reverse phase adsorption chromatography was used for determination of drug concentration. The instrument consisted of a Shimadzu LC-10AT VP pump, a SIL-10AF autoinjector, an SPD-10A UV-VIS detector, and an SCL-10A VP system controller (Shimadzu, Japan). The column was Shim-pack VP-ODS, having 4.6 mm I.D. and 150 mm bed length with adsorbent particle size 5 *μ*m (Shimadzu, Japan). The sample was prepared in methanol and 20 *μ*L was injected into the column. The column was eluted isocratically with Acetonitrile, methanol, and pH 7.4 phosphate buffer (20 : 40 : 40, v/v/v) at 1.0 mL/min. The detection wavelength was set at 275 nm [21, 24].

Calibration curve was plotted by taking concentration in range of 1–50 ng /mL with respect to peak area. A linear correlation between peak area and concentration was obtained within 2–40 ng/mL concentration range. Calibration curve equation was *y* = 48758*x* − 83696 (*R*² = 0.9972), where *x* is the concentration and *y* is the peak area.

#### 2.2.6. *In Vitro* Drug Release

Cellulose acetate synthetic membrane was used for determining the drug release. The membrane had molecular cutoff of 12000 Da. At first, the membrane was kept in physiological saline solution at 37 ± 0.5°C. It was placed on the partition inside Franz diffusion cell between donor and receptor fluid. Phosphate buffer saline pH 5.5 was filled in receptor fluid. The diameter of the cell was 3.14 cm^2^. At appropriate time, the formulation (1 g) was applied on top of the membrane. The light protection and nonocclusion were maintained throughout the study. Suitable aliquots were taken out and replaced by fresh buffer at every 2-hour interval till 24 hours. HPLC assay was used to analyze the aliquots after suitable dilution [21, 25].

#### 2.2.7. Stability Studies

The formulations were evaluated for stability by initially storing the vesicles at 4°C ± 2°C and 60 ± 5% relative humidity (RH) for 180 days and analyzing physical parameters and *in vitro* release [26].

#### 2.2.8. Preparation of Gel

Further, the nanovesicles were formulated into gel for ease in application. Carbopol 934 was dispersed in water and dispersion (1%) was prepared. The dispersion was mechanically stirred and then neutralized with triethanolamine solution (0.5%). The neutralized dispersion was kept overnight to remove any air bubble. Nanovesicles were then added to the dispersion [27].

#### 2.2.9. Viscosity

Brookfield DV III ultra V6.0 RV cone and plate rheometer (Brookfield Engineering Laboratories, Inc., Middleboro, MA, USA) was used to determine the viscosity using spindle number CPE40 at 25 ± 0.5°C [27].

#### 2.2.10. *Ex Vivo* Skin Penetration Studies

*Ex vivo* permeation study was performed using full thickness human skin obtained from plastic surgery patients. The experiment protocol was reviewed and approved by Institutional Ethical Committee, Department of Anatomy, ITS-Centre for Dental Studies and Research, Muradnagar (vide letter number PG/Research/11/04), dated 23/09/2011. The skin was cleaned with ringer solution after removing subcutaneous fatty tissues. Then it was dried and stored at −20°C packed in polyethylene bag. Prior to the study, skin sample was brought to 37°C and cleaned with ringer solution. It was then placed in donor chamber of Franz diffusion cell with epidermal side towards the donor chamber. The receptor fluid was filled with PBS (pH 5.5) to simulate the physiological environment. This setup was kept overnight for equilibration. Then, appropriate dose of the formulation (1 g, 1%) was applied. The condition of light protection and nonocclusion were maintained throughout the study. Suitable aliquots were taken out at predecided time and replaced by fresh buffer. The drug content was determined by HPLC assay.

Following the experiment, the skin was stripped ten times using scotch crystal tape. After stripping, tapes were transferred into a glass vial of suitable size according to the following plan: vial 1 = strip 1, vial 2 = strips 2-3, vial 3 = strips 4–6, and vial 4 = strips 7–10. Then the rest of epidermis was removed using surgical scalpel. Residual skin sample was homogenized in methanol and analyzed for drug content [21, 25, 28].

#### 2.2.11. *In Vivo* Skin Permeation

The experiment protocol was reviewed and approved by Institutional Animal Ethics Committee, Department of Pharmacology, ITS Paramedical College, Muradnagar (vide letter number 1044/c/07/CPCSEA-2011-MPh-07), dated 08/11/2011. Three groups of six male albino rats were used in the study. The animals were 7–9 weeks old and housed in polypropylene cages under standard laboratory conditions (temperature: 25 ± 2°C; relative humidity: 55 ± 5%), with free access to standard laboratory diet (Lipton feed, Mumbai, India) and water *ad libitum*. The animals were acclimatized for one week and 8–10-week-old rats were used for the study.

For transdermal administration, the animals were sedated with ketamine hydrochloride (75 mg/kg) and xylazine (5 mg/kg). The abdominal area was cleaned with distilled water after trimming the abdominal hairs. The treatment was applied according to the following plan with a gentle rub and held in place by open containers glued to the skin by a silicon rubber having a nominal area of 3.14 cm^2^. At appropriate time interval, 0.2 mL blood sample was taken in vacutainer tubes and processed to separate the plasma by means of centrifugation at 8000 rpm for 15 min. Plasma was stored at −21°C before performing the drug content analysis with HPLC Assay [21, 29]. One has

 Group I: CF (commercial formulation; 1 g, 1.5%; ~15 mg Aceclofenac),
 Group II: GLG-1 (500 mg, 1% ~5 mg Aceclofenac),
 Group III: PCG-1 (500 mg, 1% ~5 mg Aceclofenac).

#### 2.2.12. Anti-Inflammatory

Carrageenan induced edema model was employed for determining anti-inflammatory activity. The study protocol was designed and approved by the Institutional Animal Ethical Committee. Selected formulations (GLG-1 and PCG-1) were compared with a standard anti-inflammatory drug (Aceclofenac suspension) and a CF in four groups containing six animals in each group. Animals were fasted for 24 h before the experiment with free access to water.

Treatments were administered as per the following plan:

 Group I: Aceclofenac suspension, p.o., 20 mg/Kg,
 Group II: CF (1 g),
 Group III: GLG-1 (500 mg),
 Group IV: PCG-1 (500 mg).

Transdermal administration was kept in place by open containers glued to the abdominal skin by a silicon rubber. The untreated paw was taken as negative control. After one hour, 1% carrageenan suspension in saline was prepared and 0.1 mL was injected into right hind paw. After every hour, the paw volume was measured to yield the values for initial and at 1, 2, 3, 4, 5, and 6 h using digital plethysmograph. Percentage of inflammation was calculated by using formula given in data analysis [30].

#### 2.2.13. Skin Irritation Studies

Two groups of 6 males in each group were used. All the subjects were properly educated about the study procedure and consent forms were signed. Irritation potential was evaluated by visual observations in comparison to 5% Sodium Lauryl Sulfate (SLS) solution as positive control and untreated skin as negative control and scores were given as follows

 no reaction: 0,
 weak spotty or diffuse erythema: 1,
 weak but well perceptible erythema covering the total exposure area: 2,
 moderate erythema: 3,
 severe erythema with edema: 4,
 very severe erythema with epidermal defects: 5.

For the study, upper arm area was thoroughly examined for any irregularities and 1 g formulation was administered by gentle rubbing and held onto place with a bandage. After 24 hours, the bandage was detached and application site was cleaned with cotton. Then again application of treatment was done, for seven consecutive days. After seven days, the scores were given based on the observations [31]:

 Group I: positive control (SLS treated),
 Group II: GLG-1.

### 2.3. Data and Statistical Analysis

#### 2.3.1. *Ex Vivo* Permeation

For determination of permeation parameters, cumulative amount of drug permeated was plotted against time. The linear portion of the curve was extrapolated and projected *x*-intercept was taken as lag time (*T*_lag_). The slope of the linear region of the curve gave steady state drug flux (*J*_ss_).

Permeability coefficient through the membrane (*K*_*p*_) was calculated from steady state drug flux for administered dose of drug (*C*_*d*_) as per the following formula:

(2) $$\begin{array}{c} K_{p}=\frac{J_{\text{ss}}}{C_{d}}. \end{array}$$

Diffusion constant within the membrane (*D*; cm^2 ^h^−1^) was determined from lag time for a barrier of known thickness (*h*) [32–39]:

(3) $$\begin{array}{c} D=\frac{h^{2}}{{6\cdot t}_{\text{lag}}}. \end{array}$$

Enhancement ratio was determined from fluxes of formulations as per the following formula [40, 41]:

(4) $$\begin{array}{c} \text{ER}=\frac{\text{Flux}\;\text{of}\;\text{test}\;\text{formulation}}{\text{Flux}\;\text{of}\;\text{carbopol}\;\text{gel}\;\text{containing}\;\text{plain}\;\text{drug}}. \end{array}$$

#### 2.3.2. Pharmacokinetic Parameter

Pharmacokinetic parameters, that is, *C*_max⁡_, *T*_max⁡_, and AUC_0→*t*_, were determined from plasma concentration (*μ*g) versus time (hrs) profile. *C*_max⁡_, *T*_max⁡_ were observed directly from the profile and AUC_0→*t*_  was calculated according to linear trapezoidal method using Graph pad Prism Version 4 [29].

#### 2.3.3. Anti-Inflammatory Activity

Percentage of higher edema inhibition provided by the treatment was calculated to determine the anti-inflammatory potential as per the following formula [30]:

(5) $$\begin{array}{c} \left(\frac{T_{c}-T_{t}}{T_{c}}\right)\times 100, \end{array}$$

where *T*_*c*_ is thickness of paw in control; *T*_*t*_ is thickness of paw in treatment group.

All the experiments involving live subjects (animal or human) were done on a group of 6 whereas other studies, for example, physicochemical characterization and stability studies, were performed in triplicate. Data is expressed as mean ± S.D. Statistical analyses were performed using the Graph pad Prism Version 4 software. Statistical comparisons were made using analysis of variance (ANOVA) or the paired *t*-test, where appropriate and statistical significance was set at *P* < 0.05.

## 3. Results

### 3.1. Physicochemical Characterization

The developed formulations were characterized for physicochemical parameters, for example, size, PDI, zeta potential, entrapment efficiency, and *in vitro* drug release, since these parameters are affected by composition of the vesicles. On the other hand, these parameters significantly affect the overall effectiveness of the vesicular formulation.

**Figure 1:**
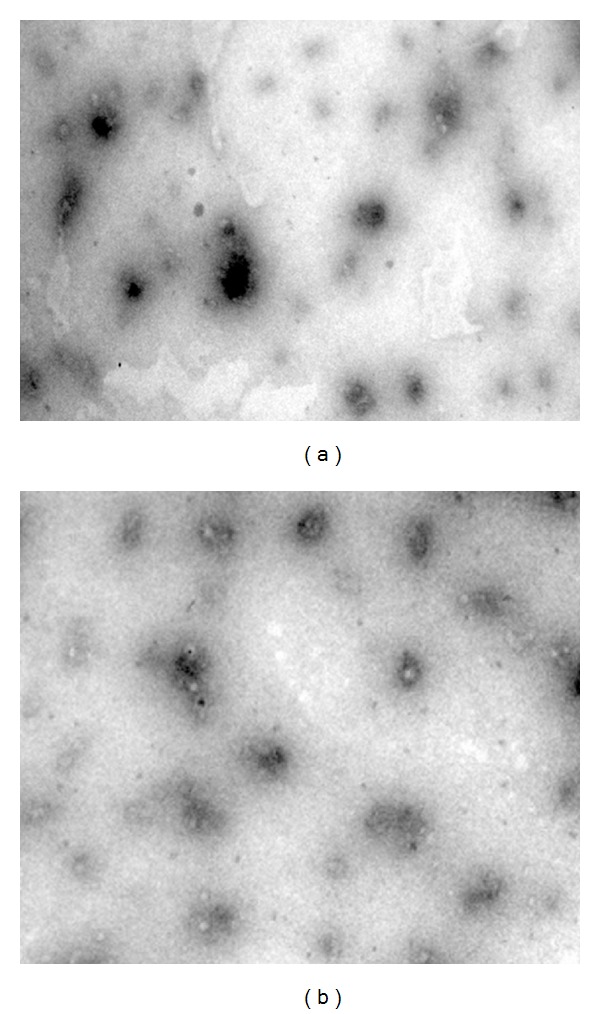
Photomicrograph of PC-1 and GL-1 (×10000) in TEM: (a) PC-1 and (b) GL-1.

Figure 1 shows round shaped vesicles approximately of 129–147 and 103–121 nm size range. The average polydispersity indices values of 0.265–0.297 and 0.132–0.153 indicate the homogeneous nature of the nanovesicle. The drug content entrapped inside the nanovesicles was in range of 26.3–47.6% and 53.4–78.9% representing a superior quantity of drug entrapped in guggul lipid nanovesicles. The zeta potential recorded for all the formulation was negative and in range of 39–42 and 16–25 mV, respectively, for PC and GL formulations (Table 2).

### 3.2. *In Vitro* Drug Release

**Figure 2:**
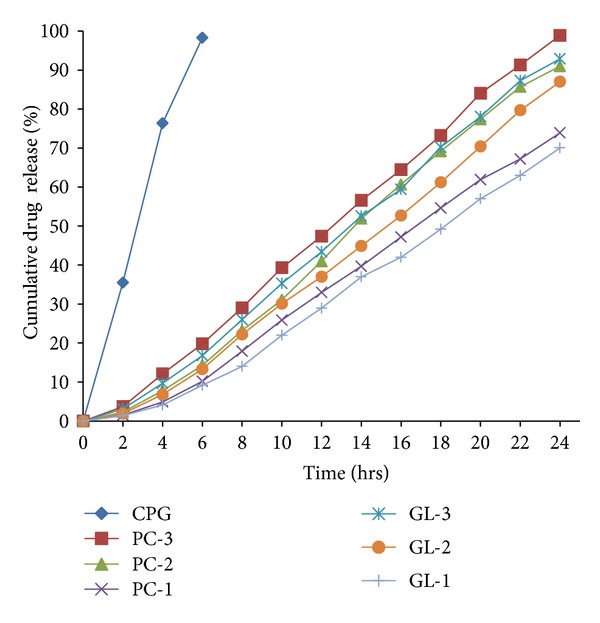
*In vitro* drug release profile of the developed nanovesicle formulations with respect to CPG.

Figure 2 shows the profile of *in vitro* drug release obtained by plotting cumulative drug release in 24 hours against time (hrs). PC-3 and GL-3 showed the highest drug release in each category and PC-1 and GL-1 showed controlled release of drug over 24 hours. Gel containing plain drug released 98.3% drug in 6 hours only.

### 3.3. Stability Studies

Based on physicochemical characterization, PC-1 and GL-1 were considered for stability evaluation at accelerated conditions for duration of 180 days at the temperature of 4°C and 25°C. The maximum damage was done by 25°C in 180 days in both types of formulations.

PC-1 was most affected by accelerated conditions. It showed 2.8 and 4.7 times value for vesicle size at 4 and 25°C after 180 days whereas PDI became 1.89 and 2.43 times at similar temperature. At 25°C, PC-1 showed considerable aggregation of vesicles after 30 days only as vesicle size and PDI are increased by 44% and 58% and entrapment efficiency decreased by 26.89%. Zeta potential increased by 21.42% after storing at 25°C for 180 days.

GL-1 showed maximum instability after 180 days as vesicle size increased by 56.19%; however, increase in vesicle size was 14% if stored at 4°C for 180 days and 3% if stored at 25°C for 30 days. Further, entrapment efficiency was decreased by 10.77% after storing at 25°C for 180 days (Table 3).

### 3.4. *Ex Vivo* Drug Permeation

**Figure 3:**
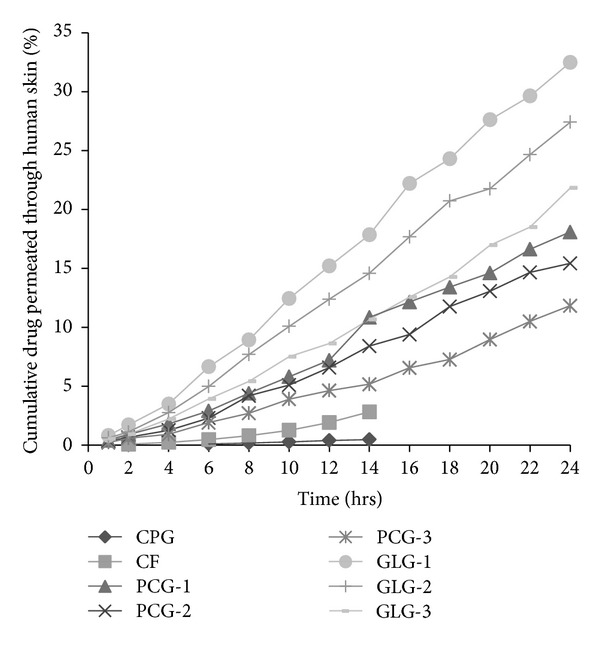
*Ex vivo* drug permeation of developed nanovesicle gels through human skin with respect to CPG and CF.

Full thickness human skin was used to study the permeation profile of the developed formulations and for computing the permeation parameters.  Figure 3 shows the cumulative amount of drug reached in receptor fluid via human skin. GLG-1 showed the maximum level of drug in receptor fluid, with the range of drug for guggul lipid formulations being 21.83–32.49 *μ*g/cm^2^. PCG formulations showed drug content in the range of 11.83–18.09 *μ*g/cm^2^. CPG showed appearance of drug by the 6th hour and most of the drug release occurring till 14 hours. CF showed appearance of drug after 2 hours and released the drug till 14 hours; however, the drug content was almost 5.97 times higher than CPG. The drug content was 69.12 and 38.48 times higher than CPG, respectively, for GLG-1 and PCG-1.

**Figure 4:**
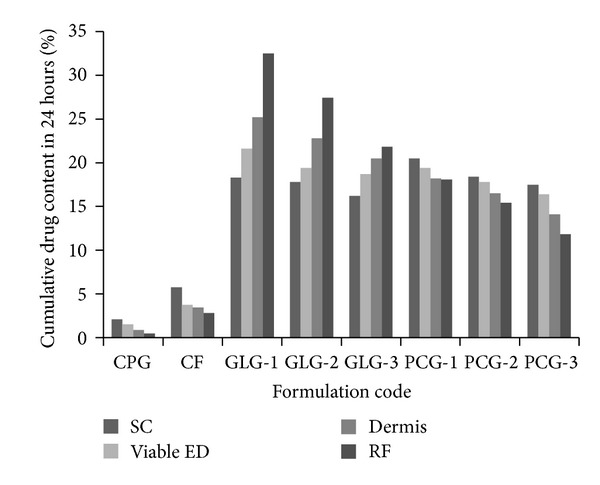
Drug deposition profile of developed nanovesicle gels in different layers of skin with respect to CPG and CF; SC: stratum corneum; VED: viable epidermis; D: dermis; RF: receptor fluid.

The total content of drug accumulated in various skin layers was determined by analyzing drug content in different strata of skin after separation of these layers by stripping.  Figure 4 illustrates a comparison of percent amount of drug accumulated in 24 hours in various levels of skin. The drug content reached up to receptor fluid through skin can be used to roughly predict the course of the formulation while being used transdermally. CPG showed less than 0.5% drug in receptor fluid. An interesting finding is that, for Guggul lipid formulations, drug content was in increasing order towards the inner side of skin layers. The drug contents in dermal layer and receptor fluid were in equilibrium.

Based on *ex vivo* skin permeation data, steady state drug flux, lag time, permeability coefficient through the skin, diffusion parameter within the skin, and enhancement ratio (in comparison to CPG) were calculated and presented in Table 4. GLG-1 showed the highest steady state drug flux (1.4295 *μ*g/cm^2^/h) and enhancement ratio (29.17) with respect to CPG (flux 0.049 *μ*g/cm^2^/h). The lag time values were in range of 1-2 hours for all the vesicle formulation showing the fast movement of drug in vesicular formulations. CPG showed a lag time value of 4.55 hours and the drug content was also very low.

### 3.5. *In Vivo* Permeation

**Figure 5:**
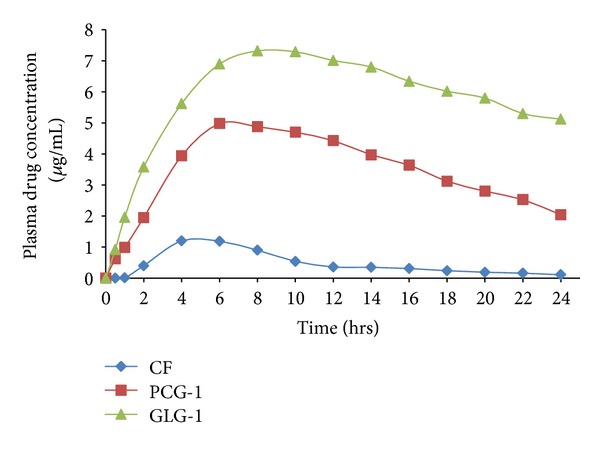
Plasma drug concentration profile of selected nanovesicle gels with respect to CF.

*In vivo* drug permeation was studied for selected formulations (GLG-1 and PCG-1) in comparison to CF. GLG-1 showed consistently increasing content of drug till 8 hours making it the  *T*_max⁡_  whereas PCG-1 and CF showed  *T*_max⁡_  at 6 and 4 hours, respectively (Figure 5). The values of *C*_max⁡_ were 4.98 and 7.32 *μ*g/mL for PCG-1 and GLG-1, respectively. AUC_0→*t*_ was 11.6 *μ*g·hr/mL, 83.9 *μ*g·hr/mL, and 141.2 *μ*g·hr/mL for CF (applied dose 1 g; ~15 mg Aceclofenac), PCG-1, and GLG-1 (applied dose 500 mg; ~5 mg Aceclofenac) respectively (Table 5).

### 3.6. Anti-Inflammatory Activity

**Figure 6:**
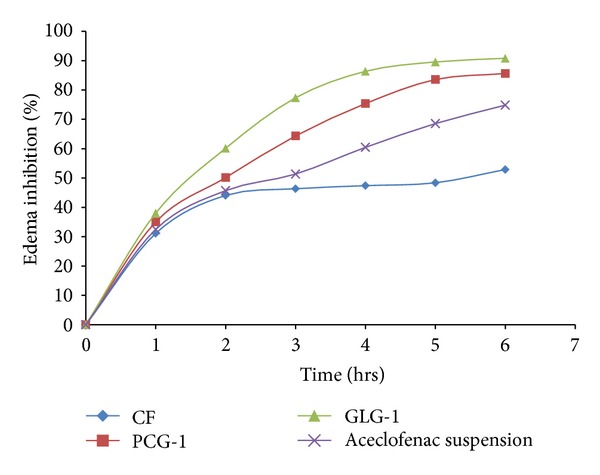
% edema inhibition provided by selected formulations (GLG-1, PCG-1, CF, and Aceclofenac).

Carrageenan induced paw edema method was used for determining edema inhibition provided by selected formulation in comparison to CF and a standard treatment, oral Aceclofenac. In the initial hour, edema inhibition was the same for all the treatment; however, in second phase of edema production, GLG-1 provided 90.81% edema inhibition closely followed by PCG-1 at 85.62%. Standard treatment produced 74.84% edema inhibition in 6 hours whereas CF afforded 52.89% edema inhibition. It was special to note that PCG-1 afforded almost the same edema inhibition which might be due to topical nature of inflammation present in this kind of *in vivo* activity model (Figure 6).

### 3.7. Irritation Potential

No group showed any severe irritation except the group treated with SLS.

## 4. Discussion

The formulations PC-1 and GL-1 showed optimum physicochemical parameters. Smaller vesicle size produces larger surface area and appropriately charged zeta potential keeps them away so that the vesicle formulations remain stable.

The entrapment efficiency increased with increase in lipid content. Cholesterol is present in the same concentration in all the vesicle formulation. It is also reported to have an additive effect on increasing the drug entrapment.

Zeta potential is an important parameter owing to the role of surface charge in stabilization of the vesicle formulation; however, it may be affected by vesicle size, surface area, spatial localization of various components, and their state of ionization at the pH of application site. DCP is the only compound carrying electric charge and the difference in vesicle size is also nonsignificant.

The drug release profile shows that the highest drug releases were found in PC-3 and GL-3, having lower concentration of lipid. PC-1 and GL-1 showed the lowest drug release in their category having higher lipid content. PC and GL vesicles contain gradually decreasing fraction of PC, and guggul lipid, respectively. The content of cholesterol and DCP remained the same. PC is a phospholipid and forms bilayer in aqueous medium. Cholesterol seals the gap in PC membrane thereby used as an integral part of liposome composition to increase the stability. It increases drug entrapment and decreases drug release. The PC-1 and GL-1 were selected based on their physicochemical parameters and stability profile.

The selected formulations showed the difference in stability evaluation as PC-1 showed severe instability at higher temperature range in short duration and even at 4°C in 6 months. Temperature and time period both have shown detrimental effect on PC-1. Phosphatidylcholine is prone to hydrolysis and oxidation during storage producing lysolecithin. The presence of lysolecithin in lipid bilayers greatly enhances the permeability of liposomes. This is evident in reduction of time for drug release in PC-1 after storage at higher temperature. The similar event occurred at 4°C albeit after a longer time period. GL-1 showed commendable stability at 4°C; however, even at 25°C the stability is acceptable. Guggul lipid is composed of guggulsterones which are isomeric compounds of steroids category. The chemical structure is similar to cholesterol minus the side chain of cholesterol. The structure of guggulsterone is planar. The authors hypothesize the molecule by molecule stacking of guggulsterone and cholesterol considering steric hindrance due to cholesterol side chain. Further, due to this kind of membrane structure, more content of drug is retained even after 24 hours.

The drug content in receptor fluid was almost double in GLG formulation in comparison to the PCG formulations of the same ratio. An interesting finding is that for PCG formulations content of drug was higher in upper layers of skin. This is in accordance with the previous findings about rupture of PC liposome in upper layers of skin and drug deposition in superficial layers. In Guggul lipid vesicles, drug content in receptor fluid was in equilibrium with drug content in dermis which means that guggul lipid vesicles enhance the drug permeation inside the skin.

*In vivo* study and anti-inflammatory activity replicate the results of *ex vivo* study and guggulsterone itself possess anti-inflammatory activity.

## 5. Conclusion

The study revealed that guggul lipid vesicles showed the optimum physical parameters and permeation profile; however, more significantly it shows good stability profile over PC liposomes. The most promising formulation was found to be GLG-1 with a composition of 7 : 3 : 1 (guggul lipid : cholesterol : DCP). We suggest that guggul lipid vesicles would be beneficial for transdermal drug delivery.

## Figures and Tables

**Table 1:** Composition of the nanovesicle formulations (lipid drug ratio = 3 : 1).

S. No.	Formulation code	Composition	Molar ratio (% w/w)
1.	PC-1	PC/Chol/DCP	7/3/1
2.	PC-2	PC/Chol/DCP	5/3/1
3.	PC-3	PC/Chol/DCP	3/3/1
4.	GL-1	GL/Chol/DCP	7/3/1
5.	GL-2	GL/Chol/DCP	5/3/1
6.	GL-2	GL/Chol/DCP	3/3/1

PC: Phosphatidylcholine nanovesicle; GL: Guggul Lipid nanovesicle; Chol: cholesterol; DCP: Dicetyl phosphate.

**Table 2:** Physicochemical evaluation of developed nanovesicle formulations.

Formulation code	Size* (nm)	PDI*	*ζ* potential* (mV)	Entrapment efficiency* (%)	Viscosity^†^ (Cps)
PC-1	147 ± 2.5	0.297 ± 0.001	−42 ± 1.2	47.6 ± 1.8	17760 ± 5.84
PC-2	136 ± 1.6	0.289 ± 0.003	−40 ± 1	34.2 ± 1.4	17617 ± 4.86
PC-3	129 ± 2.4	0.265 ± 0.007	−39 ± 0.8	26.3 ± 2.6	17264 ± 4.98
GL-1	121 ± 1.1	0.153 ± 0.004	−25 ± 1.1	78.9 ± 1.1	18321 ± 3.65
GL-2	114 ± 1.3	0.143 ± 0.008	−19 ± 1.3	67.7 ± 3.1	18102 ± 2.79
GL-2	103 ± 3.5	0.132 ± 0.002	−16 ± 1.9	53.4 ± 1.2	17745 ± 3.23

All data expressed as mean ± S.D.; *n* = 3; *P* ≤ 0.05.

**Table 3:** Physicochemical characterization of selected nanovesicle formulations (PC-1 and GL-1) after stability studies.

Parameters	Formulation code	0th Day	30th Day		90th Day		180th Day	
			4°C	25°C	4°C	25°C	4°C	25°C
Size (nm)	PC-1	147 ± 2.5	163 ± 1.3	212 ± 4.2	285 ± 2.4	346 ± 2.8	412 ± 3.7	698 ± 4.7
	GL-1	121 ± 1.1	122 ± 1.5	125 ± 1.7	129 ± 2.8	143 ± 2.5	138 ± 1.6	189 ± 1.9

PDI	PC-1	0.297 ± 0.001	0.351 ± 0.003	0.472 ± 0.007	0.449 ± 0.002	0.548 ± 0.003	0.562 ± 0.009	0.723 ± 0.007
	GL-1	0.153 ± 0.004	0.167 ± 0.002	0.171 ± 0.001	0.179 ± 0.001	0.185 ± 0.007	0.182 ± 0.005	0.198 ± 0.001

*ζ* potential (mV)	PC-1	−42 ± 1.2	−43 ± 1.8	−44 ± 1.3	−46 ± 3.1	−48 ± 3.5	−48 ± 2.9	−51 ± 2.6
	GL-1	−25 ± 1.1	−26 ± 1.7	−27 ± 2.9	−26 ± 2.9	−28 ± 1.6	−27 ± 1.9	−29 ± 2.4

Entrapment efficiency (%)	PC-1	47.6 ± 1.8	46.8 ± 1.3	34.8 ± 2.1	38.2 ± 1.5	28.1 ± 2.6	32.6 ± 2.1	17.2 ± 2.2
	GL-1	78.9 ± 1.1	78.4 ± 1.3	77.3 ± 1.5	77.6 ± 1.5	73.2 ± 1.4	74.1 ± 1.8	70.4 ± 1.7

*In vitro* % Cumulative drug release	PC-1	73.91 ± 1.4 **(in 24 hrs)**	79.2 ± 1.9 **(in 18 hrs)**	91.4 ± 2.2 **(in 18 hrs)**	85.5 ± 2.8 **(in 20 hrs)**	94.3 ± 2.3 **(in 14 hrs)**	93.7 ± 1.4 **(in 12 hrs)**	95.3 ± 1.9 **(in 8 hrs)**
	GL-1	70.06 ± 1.3 **(in 24 hrs)**	72.4 ± 1.9 **(in 24 hrs)**	75.6 ± 1.5 **(in 24 hrs)**	78.8 ± 2.2 **(in 24 hrs)**	81.3 ± 1.9 **(in 24 hrs)**	89.5 ± 1.6 **(in 24 hrs)**	95.2 ± 1.4 **(in 24 hrs)**

All data expressed as mean ± S.D.; *n* = 3; *P* ≤ 0.05.

**Table 4:** *Ex vivo* permeation parameters for developed nanovesicle gels (PCG and GLG) with respect to CPG and CF.

Formulation code	Flux (*μ*g/cm^2^/h)	Lag time (hrs)	Permeability coefficient (cm/h)	Distribution coefficient (cm^2^/h × 10^−3^)	Enhancement ratio
CPG	0.049	4.55	4.9 × 10^−6^	0.769	1
CF	0.2218	3.2	2.218 × 10^−5^	1.093	4.52
PCG-1	0.806	1.675	8.06 × 10^−5^	2.08	16.44
PCG-2	0.6997	1.875	6.997 × 10^−5^	1.866	14.27
PCG-3	0.4941	1.95	4.941 × 10^−5^	1.794	10.08
GLG-1	1.4295	1.15	1.4295 × 10^−4^	3.043	29.17
GLG-2	1.1928	1.35	1.1928 × 10^−4^	2.59	24.34
GLG-3	0.9047	1.55	9.047 × 10^−5^	2.258	18.46

**Table 5:** Pharmacokinetic parameters of selected nanovesicle gel formulations (PCG-1 and GLG-1) with respect to CF.

Formulation code	*C*_max⁡_ (*μ*g/mL)	*T*_max⁡_ (hrs)	AUC (*μ*g·hr/mL)
CF*	1.2 ± 0.023	4	11.6
PCG-1	4.98 ± 0.95	4	83.9
GLG-1^†^	7.32 ± 0.29	8	141.2

*1 g formulation equivalent to 15 mg Aceclofenac.

^†^500 mg formulation equivalent to 5 mg of Aceclofenac.

All data expressed as mean ± S.D.; *n* = 6; (*P* ≤ 0.05).

## References

[1] Kostarelos K (2003). Rational design and engineering of delivery systems for therapeutics: biomedical exercises in colloid and surface science. *Advances in Colloid and Interface Science*.

[2] Nasr M, Mansour S, Mortada ND, Elshamy AA (2008). Vesicular aceclofenac systems: a comparative study between liposomes and niosomes. *Journal of Microencapsulation*.

[3] Blume G, Cevc G (1992). Drug-carrier and stability properties of the long-lived lipid vesicles, cryptosomes, in vitro and in vivo. *Journal of Liposome Research*.

[4] Nieuwenhuyzen WV, Tomás MC (2008). Update on vegetable lecithin and phospholipid technologies. *European Journal of Lipid Science and Technology*.

[5] Kontogiannopoulos KN, Assimopoulou AN, Dimas K, Papageorgiou VP (2011). Shikonin-loaded liposomes as a new drug delivery system: physicochemical characterization and in vitro cytotoxicity. *European Journal of Lipid Science and Technology*.

[6] Gaur PK, Mishra S, Gupta VB, Rathod MS, Purohit S, Savla BA (2010). Targeted drug delivery of Rifampicin to the lungs: formulation, characterization, and stability studies of preformed aerosolized liposome and in situ formed aerosolized liposome. *Drug Development and Industrial Pharmacy*.

[7] Nieuwenhuyzen WV, Szuhaj BF (1998). Effects of lecithins and proteins on the stability of emulsions. *Lipid/Fett*.

[8] Riaz M (1995). Stability and uses of liposomes. *Pakistan Journal of Pharmaceutical Sciences*.

[9] Honeywell-Nguyen PL, Bouwstra JA (2005). Vesicles as a tool for transdermal and dermal delivery. *Drug Discovery Today*.

[10] Carneiro R, Salgado A, Raposo S (2011). Topical emulsions containing ceramides: effects on the skin barrier function and anti-inflammatory properties. *European Journal of Lipid Science and Technology*.

[11] Liu DZ, Chen WY, Tasi LM, Yang SP (2000). Microcalorimetric and shear studies on the effects of cholesterol on the physical stability of lipid vesicles. *Colloids and Surfaces A*.

[12] Ahmad MU, Ali SM, Ahmad A, Sheikh S, Ahmad I (2010). Guggullipid derivatives: synthesis and applications. *Chemistry and Physics of Lipids*.

[13] Shen T, Li GH, Wang XN, Lou HK (2012). The genus Commiphora: a review of its traditional uses, phytochemistry and pharmacology. *Journal of Ethnopharmacology*.

[14] Musharraf SG, Iqbal N, Gulzar U, Ali A, Choudhary MI, Atta-ur-Rahman A (2011). Effective separation and analysis of E- and Z-guggulsterones in Commiphora mukul resin, guggulipid and their pharmaceutical product by high performance thin-layer chromatography-densitometric method. *Journal of Pharmaceutical and Biomedical Analysis*.

[15] Legrand E (2004). Aceclofenac in the management of inflammatory pain. *Expert Opinion on Pharmacotherapy*.

[16] Şeyda AA, Figen T (2010). A nonsteroidal antiinflammatory drug: aceclofenac. *FABAD Journal of Pharmaceutical Sciences*.

[17] Shavi GV, Nayak U, Averineni RK (2009). Multiparticulate drug delivery system of aceclofenac: development and in vitro studies. *Drug Development and Industrial Pharmacy*.

[18] Insel PA, Gilman AG, Rall T, Nies A, Taylor P (1990). Analgesic-antipyretics and antiinflammatory agents: drugs employed in the treatment of rheumatoid arthritis and gout. *Goodman and Gilman’s the Pharmacological Basis of Therapeutics*.

[19] Alvarez-Larena A, Piniella JF, Carrasco E, Ginebreda A, Julia S, Germain G (1992). Crystal structure and spectroscopic study of 2-[(2,6-dichlorophenyl)amino]phenylacetoxyacetic acid (Aceclofenac). *Journal of Crystallographic and Spectroscopic Research*.

[20] Sinico C, Manconi M, Peppi M, Lai F, Valenti D, Fadda AM (2005). Liposomes as carriers for dermal delivery of tretinoin: in vitro evaluation of drug permeation and vesicle-skin interaction. *Journal of Controlled Release*.

[21] Gaur PK, Purohit S, Kumar Y, Mishra S, Bhandari A (2014). Preparation, characterization and permeation studies of a nanovesicular system containing diclofenac for transdermal delivery. *Pharmaceutical Development and Technology*.

[22] Paradissis A, Hatziantoniou S, Georgopoulos A, Demetzos C (2005). Lipid analysis of Greek broad bean oil: preparation of liposomes and physicochemical characterization. *European Journal of Lipid Science and Technology*.

[23] Touitou E, Dayan N, Bergelson L, Godin B, Eliaz M (2000). Ethosomes-novel vesicular carriers for enhanced delivery: characterization and skin penetration properties. *Journal of Controlled Release*.

[24] Lee J, Lee Y, Kim J, Yoon M, Young WC (2005). Formulation of microemulsion systems for transdermal delivery of aceclofenac. *Archives of Pharmacal Research*.

[25] Dragicevic-Curic N, Scheglmann D, Albrecht V, Fahr A (2008). Temoporfin-loaded invasomes: development, characterization and in vitro skin penetration studies. *Journal of Controlled Release*.

[26] Ishida T, Takanashi Y, Doi H, Yamamoto I, Kiwada H (2002). Encapsulation of an antivasospastic drug, fasudil, into liposomes, and in vitro stability of the fasudil-loaded liposomes. *International Journal of Pharmaceutics*.

[27] El-Leithy ES, Shaker DS, Ghorab MK, Abdel-Rashid RS (2010). Evaluation of mucoadhesive hydrogels loaded with diclofenac sodium-chitosan microspheres for rectal administration. *AAPS PharmSciTech*.

[28] Manconi M, Caddeo C, Sinico C (2011). Ex vivo skin delivery of diclofenac by transcutol containing liposomes and suggested mechanism of vesicle-skin interaction. *European Journal of Pharmaceutics and Biopharmaceutics*.

[29] Akhter S, Jain GK, Ahmad FJ (2008). Investigation of nanoemulsion system for transdermal delivery of domperidone: ex vivo and in vivo studies. *Current Nanoscience*.

[30] Manosroi A, Jantrawut P, Manosroi J (2008). Anti-inflammatory activity of gel containing novel elastic niosomes entrapped with diclofenac diethylammonium. *International Journal of Pharmaceutics*.

[31] Patel NA, Patel NJ, Patel RP (2009). Formulation and evaluation of curcumin gel for topical application. *Pharmaceutical Development and Technology*.

[32] Higuchi T (1960). Physical chemical analysis of percutaneous absorption process from creams and ointments. *Journal of the Society of Cosmetic Chemists*.

[33] Aguiar AJ, Weiner MA (1969). Percutaneous absorption studies of chloramphenicol solutions. *Journal of Pharmaceutical Sciences*.

[34] Dugard PH, Marzulli FN, Maibach HI (1976). Advanccs in modern toxicology. *Dermatotoxicology and Pharmacology*.

[35] Durrheim H, Flynn GL, Higuchi WI, Behl CR (1980). Permeation of hairless mouse skin. I: experimental methods and comparison with human epidermal permeation by alkanols. *Journal of Pharmaceutical Sciences*.

[36] Haigh JM, Beyssac E, Chanet L, Aiache J-M (1998). In vitro permeation of progesterone from a gel through the shed skin of three different snake species. *International Journal of Pharmaceutics*.

[37] Kobayashi N, Saitoh I (1999). Development of a test method for in vitro drug release from soluble and crystal dispersion type ointments. *Chemical and Pharmaceutical Bulletin*.

[38] Vasiljevic D, Parojcic J, Primorac M, Vuleta G (2006). An investigation into the characteristics and drug release properties of multiple W/O/W emulsion systems containing low concentration of lipophilic polymeric emulsifier. *International Journal of Pharmaceutics*.

[39] Furuishi T, Io T, Fukami T, Suzuki T, Tomono K (2008). Formulation and in vitro evaluation of pentazocine transdermal delivery system. *Biological and Pharmaceutical Bulletin*.

[40] Mishra D, Garg M, Dubey V, Jain S, Jain NK (2007). Elastic liposomes mediated transdermal delivery of an anti-hypertensive agent: propranolol hydrochloride. *Journal of Pharmaceutical Sciences*.

[41] Jukanti R, Sheela S, Bandari S, Veerareddy PR (2011). Enhanced bioavailability of exemestane via proliposomes based transdermal delivery. *Journal of Pharmaceutical Sciences*.

